# Sequence-dependent fusion dynamics and physical properties of DNA droplets†

**DOI:** 10.1039/d3na00073g

**Published:** 2023-02-14

**Authors:** Yusuke Sato, Masahiro Takinoue

**Affiliations:** a Department of Computer Science, Tokyo Institute of Technology 4259, Nagatsuta-cho, Midori-ku Yokoham Kanagawa 226-8502 Japan takinoue@c.titech.ac.jp; b Department of Intelligent and Control Systems, Kyushu Institute of Technology 680-4 Kawazu, IIzuka Fukuoka 820-8502 Japan ysato@ics.kyutech.ac.jp; c Living Systems Materialogy (LiSM) Research Group, International Research Frontiers Initiative (IRFI), Tokyo Institute of Technology 4259, Nagatsuta-cho, Midori-ku Yokohama 226-8501 Japan

## Abstract

Liquid–liquid phase separation (LLPS) of biopolymer molecules generates liquid-like droplets. Physical properties such as viscosity and surface tension play important roles in the functions of these droplets. DNA-nanostructure-based LLPS systems provide useful model tools to investigate the influence of molecular design on the physical properties of the droplets, which has so far remained unclear. Herein, we report changes in the physical properties of DNA droplets by sticky end (SE) design in DNA nanostructures. We used a Y-shaped DNA nanostructure (Y-motif) with three SEs as a model structure. Seven different SE designs were used. The experiments were performed at the phase transition temperature where the Y-motifs self-assembled into droplets. We found that the DNA droplets assembled from the Y-motifs with longer SEs exhibited a longer coalescence period. In addition, the Y-motifs with the same length but different sequence SEs showed slight variations in the coalescence period. Our results suggest that the SE length greatly affected the surface tension at the phase transition temperature. We believe that these findings will accelerate our understanding of the relationship between molecular design and the physical properties of droplets formed *via* LLPS.

## Abstract 2

Physical properties of DNA droplets formed by liquid–liquid phase separation of DNA nanostructures were investigated at a phase transition temperature. The surface tension was strongly influenced by the design of sticky ends.

## Graphical abstract

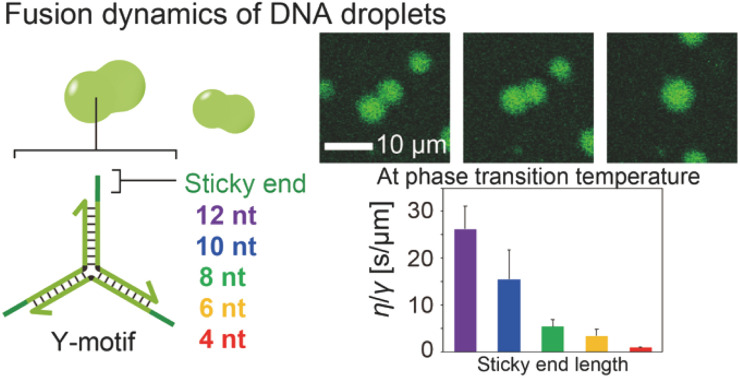

## 1 Introduction

Liquid–liquid phase separation (LLPS) of biopolymer molecules to generate hydrogel- or liquid-like molecular condensates has recently emerged as a focal point of biological research.^1,2^ The generation of these condensates underpins various cellular processes, such as the formation of membraneless organelles^3^ and remodelling of cellular membranes.^4,5^ To elucidate the molecular mechanisms underlying biopolymer LLPS, research has been performed in various fields, including biophysics,^6^ chemical biology,^7^ and soft matter physics.^8^

Understanding not only the formation mechanism but also the physical properties of these condensates is crucial because the physical properties govern their behaviour, particularly in the case of droplet-like structures. The surface tension and viscosity of the droplets affect the growth of membraneless organelles^9^ and their molecular exchange rates with bulk solution.^10^*In vitro* experiments have demonstrated that the physical properties are strongly dependent on the ionic conditions and charged molecules in solution.^11–14^ For example, Brangwynne *et al.* reported that nucleoli behave as liquid-like droplets and exhibit ATP-dependent viscosity.^13^ In addition, Alshareedah *et al.* reported that the viscosity and surface tension of polypeptide/nucleic acid complexes display NaCl concentration dependence.^14^ These findings confirm that electrostatic interactions play an essential role in the physical properties of droplets formed by LLPS. Although some studies have proposed design rules for modulating droplet formation by charged polymers^15^ and reported changes in the viscoelasticity of peptide–RNA condensates by varying the amino acid sequence,^16^ physical property regulation by molecular design remains challenging owing to the intrinsic lack of specificity of electrostatic interactions and the complexity of amino acid interactions. An LLPS system based on designable/predictable molecular interactions could provide a means to precisely control the physical properties of droplets.

The self-assembly of sequence-designed DNAs has been applied to construct tuneable LLPS systems by exploiting base-specific interactions.^17–28^ Our recent study showed that the self-assembly of DNA nanostructures *via* sticky end (SE) interactions displayed LLPS, leading to the formation of micrometre-sized droplets or hydrogels depending on the solution temperature.^27^ A major advantage of DNA-based LLPS systems is that the behaviour and dynamics of the DNA droplets can be regulated by judicious design of the base sequences, which enabled us to realize designable dynamic behaviour, such as selective and exclusive droplet fusion, droplet fission, and selective protein capture by the droplets.^27^ Moreover, the physical properties of hybridization-based DNA hydrogels can be tuned by varying the nanostructure design^29,30^ and ambient temperature.^31,32^ The physical properties of DNA droplets are also dependent on the salt conditions, and a higher salt (NaCl) concentration was found to increase the droplet viscosity and surface tension at room temperature (20 °C).^23^ On the other hand, at the phase transition temperature between the monomer-dispersed and droplet formation phases, the liquid-like properties became remarkable.^27^ However, the influence of the SE design on the physical properties of DNA droplets at such higher temperatures (phase transition temperatures) is still not well understood.

In this study, we examined how the SE design affects the physical properties of DNA droplets at elevated temperature. The experiments were performed at the phase transition temperature between the dispersed and droplet formation phases of DNA nanostructures. We focused on the fusion dynamics of the droplets, which reflect the viscosity and surface tension, and found that DNA droplets composed of DNA nanostructures with longer SE sequences took longer to undergo complete fusion. In addition, the mobility of the DNA nanostructures in the DNA droplets did not display a significant difference. We deduced that at the phase transition temperature, the SE sequence design (nucleotide length) exerts a large influence on the surface tension of DNA droplets but not the viscosity, whereas the SE number per DNA nanostructure affects both. Our results serve to elucidate how the physical properties of macromolecular droplets are determined and can be tuned by a molecular design approach.

## 2 Materials and methods

### 2.1. DNA nanostructure design

The DNA sequences were designed and analysed using the web software NUPACK^33^ and DINAMelt.^34^ We designed Y-shaped DNA nanostructures (hereinafter referred to as Y-motifs), which were assembled from three different single-stranded DNAs (ssDNAs).^27^ These Y-motifs can self-assemble into macromolecular structures *via* SE interactions. Seven different types of SE were designed: five different SE lengths (4, 6, 8, 10, or 12 nucleotides (nt)) and two different sequences of 6 nt (three variations of 6 nt in total; referred to as 6 nt_A, 6 nt_B, and 6 nt_C). The DNAs were purchased from Eurofins Genomics (Tokyo, Japan) and were of oligonucleotide purification cartridge grade. DNAs were stored in ultrapure water at a concentration of 100 μM and −20 °C until use. All DNA sequences used in this work are listed in Table S1.†

### 2.2. Preparation of the observation chamber

A glass observation chamber was prepared as described in our previous study.^27^ Briefly, oxygen-plasma-cleaned glass slides (30 mm × 40 mm) were treated with 5% (w/v) bovine serum albumin (BSA) in 20 mM Tris–HCl (pH 8.0). After the treatment, the slides were washed with ultrapure water and dried under a flow of air. The BSA-coated glass slides and coverslips (18 mm × 18 mm) were assembled using double-sided tape.

### 2.3. Sample preparation

DNA strands were mixed in a test tube at 5 μM each in buffer containing 20 mM Tris–HCl (pH 8.0) and 350 mM NaCl. Note that fluorescently labelled (6-carboxyfluorescein, FAM) DNA was added at 10% molar concentration, instead of non-labelled DNA.

### 2.4. Observation setup

The sample solution was loaded into the slit between the BSA-coated glass (bottom side) and coverslips (top side). The edges of the coverslips were coated with resin. The chamber was further coated with mineral oil (Nacalai Tesque, Kyoto, Japan) using a bank made of a silicone sheet to avoid evaporation during the observation.

### 2.5. Visualization

The chamber containing the sample solution was placed on a temperature control system (Linkam, Hukuoka, Japan and TPi-110RX, Tokai Hit, Japan), which was positioned on the stage of a confocal laser scanning microscope (CLSM; FV1000, Olympus, Tokyo, Japan). An objective lens with 20× magnification and 0.45 numerical aperture (LUCPLFLN, Olympus, Tokyo, Japan) was used. A 473 nm laser was used to excite FAM. The temperature was initially set to 85 °C and the sample was allowed to equilibrate for 3 min. The temperature was then decreased at a rate of −1 °C min^−1^ until droplets started to form. In the different SE lengths, the droplet formation typically occurred at 70, 61, 63, 48, and 46 °C for 12 nt, 10 nt, 8 nt, 6 nt_A, and 4 nt, respectively.^27^ For 6 nt_B and 6 nt_C it occurred at 49 and 43 °C, respectively. Sequential images were then acquired at a rate of 1 frame every 5 s to visualize the droplets.

### 2.6. Analysis of fusion dynamics

The obtained images showing the fusion of the DNA droplets were analysed using custom-written code. First, the images were binarized to remove the background signal. Then, the boundary of the droplets undergoing fusion was fitted as an ellipse. Finally, long and short axis values were obtained to evaluate the aspect ratio over time. The resulting values of the aspect ratio over time were fitted using an exponential curve:

1 *f*(*t*) = 1 + *A* exp(−*t*/*τ*_fusion_)

where *A* is a fitting parameter related to the initial aspect ratio and *τ*_fusion_ is the characteristic time.

### 2.7. Diffusion coefficient measurements

The diffusion coefficients of the Y-motifs in the DNA droplets were measured by fluorescence recovery after photobleaching (FRAP) experiments. The FRAP experiments were performed for DNA droplets over 10 μm in diameter at a temperature of 1 °C below the phase transition temperature. The frame interval was set to 1 s, and five frames were obtained before bleaching. Then, a circular region of interest (ROI) at the centre of the droplets was bleached for 1.5 s. After bleaching, the recovery of the fluorescence intensity at the ROI and a reference region (non-bleached region in the droplets) was monitored over time. The fluorescence intensities of the ROI and reference region were obtained using the FluoView software (version 4.0, Olympus, Tokyo, Japan). The fluorescence intensity of the ROI was normalized using the intensity of the reference region based on previously reported methods^35,36^ with some modifications. The normalized intensity data were fit to the following equation:2
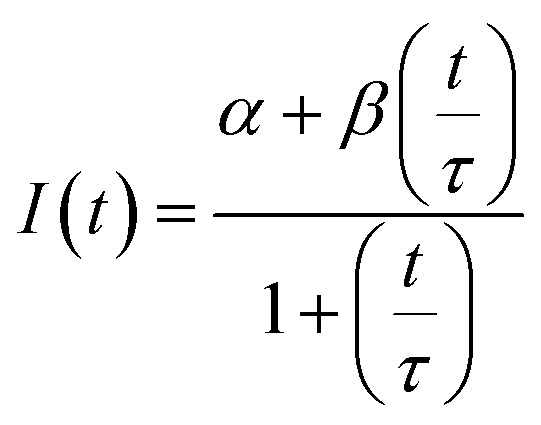
where *α* and *β* are constant terms and *τ* is the recovery time constant. The apparent diffusion coefficient (*D*_apparent_) was calculated using the following equation:^35^

3 *D*_apparent_ = *r*^2^/*τ*

where *r* is the radius of the ROI.

## 3 Results

**Fig. 1:**
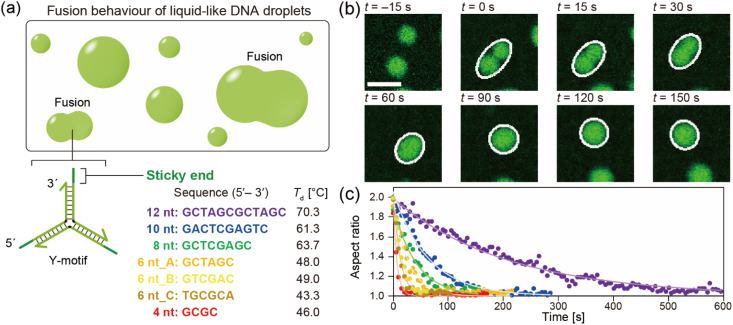
Fusion behaviour of DNA droplets. (a) Schematic diagrams showing the fusion of DNA droplets and the DNA nanostructure design. The DNA droplets were formed by the self-assembly of Y-shaped DNA nanostructures (Y-motifs) *via* the interaction of SEs. Seven SEs of different designs with different state-change temperatures between the dispersed and droplet-like states (*T*_d_) were used in this study. (b) Representative sequential images of the fusion process for DNA droplets composed of the Y-motif with an SE length of 8 nt, obtained at 63 °C. Once different droplets collided with one another, they began to fuse and eventually became a larger DNA droplet. The white ellipses indicate the fitting results used to analyse the aspect ratio of the droplets undergoing fusion. Scale bar: 10 μm. (c) Representative aspect ratios of the DNA droplets with different SE designs over time. The purple, blue, green, yellow, light yellow, dark yellow, and red colours correspond to SE designs of 12 nt, 10 nt, 8 nt, 6 nt_A, 6 nt_B, 6 nt_C, and 4 nt, respectively. The closed circles and lines indicate the experimental values and fitting curves, respectively. Note that the visualizations were performed at each *T*_d_ (shown in panel (a)) for different SE designs.

The DNA droplets were formed by the self-assembly of the Y-motifs (Fig. 1a). We previously reported that the Y-motifs formed liquid-like droplets *via* LLPS in a specific temperature range that was lower than the state-change temperature between the dispersed and droplet-like states (*T*_d_; in this paper, we refer to it as the phase transition temperature) and higher than the state-change temperature between the droplet-like and gel states (*T*_g_).^27^ We prepared seven types of Y-motifs with different SE designs to examine the influence of the SE design on the fusion dynamics. Fig. 1b presents representative sequential microscopic images showing the fusion process for the DNA droplets composed of the Y-motif with an SE length of 8 nt. The FAM-labelled Y-motifs were visualized using the CLSM. When two DNA droplets collided by Brownian motion (Fig. 1b, *t* = 0 s), their fusion was observed. The completion of this process required up to several minutes (Fig. 1b, *t* = 150 s). Similar fusion behaviour was observed for the droplets composed of the Y-motifs with all SE designs at *T*_d_ (Fig. S1†). Note that Y-motifs with longer SEs tended to have higher *T*_d_ values: 70.3 ± 1.5, 61.3 ± 0.6, 63.7 ± 0.6, 48.0 ± 1.2, and 46.0 ± 0.6 °C for SE lengths of 12, 10, 8, 6, and 4 nt, respectively (Fig. 1a).^27^ The *T*_d_ of 6 nt_B and 6 nt_C were 49.0 ± 0.0 and 43.3 ± 1.2 °C.

To evaluate the influence of the SE design on the fusion dynamics, the velocity of coalescence was compared. For this analysis, the droplets undergoing fusion were fitted as an ellipse at each time point as depicted in Fig. 1b (white ellipses) and the aspect ratio of these ellipses over time was measured. The measurement results revealed that droplets composed of Y-motifs with longer SEs tended to require longer to complete the fusion (Fig. 1c and S2†). In addition, 6 nt_B and 6 nt_C showed slightly different fusion dynamics to 6 nt_A, although their sequence length was the same as for 6 nt_A (Fig. 1c and S2†). The changes in the aspect ratios over time were well fitted by exponential curves (solid lines in Fig. 1c).

**Fig. 2:**
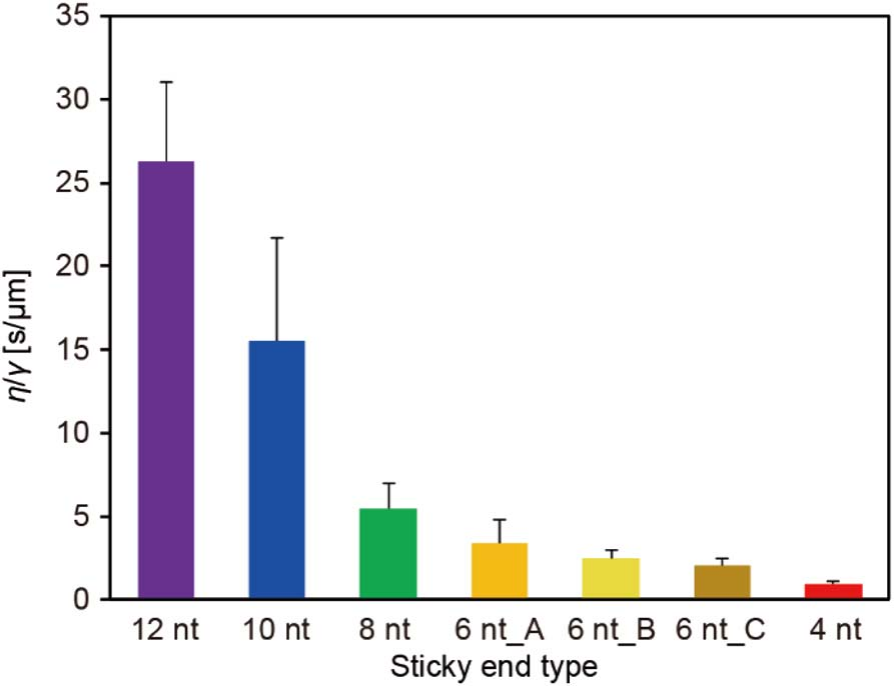
Inverse capillary velocities of DNA droplets composed of Y-motifs with various SE designs at *T*_d_ (70, 61, 63, 48, 49, 43, and 46 °C for 12 nt, 10 nt, 8 nt, 6 nt_A, 6 nt_B, 6 nt_C, and 4 nt). Error bars indicate standard deviations (*n* = 12, 13, 13, 12, 19, 14, and 14 for SE designs of 12 nt, 10 nt, 8 nt, 6 nt_A, 6 nt_B, 6 nt_C, and 4 nt, respectively).

The ratio of the viscosity and surface tension of the droplets, which is known as the inverse capillary velocity, was estimated from the relationship *τ*_fusion_ ≈ (*η*/*γ*)·*l*,^13^ where *η* is the viscosity, *γ* is the surface tension, and *l* is the characteristic length of the DNA droplet. The characteristic time *τ*_fusion_ was obtained from the curve fitting, and *l* was defined as [{(long axis) − (short axis)} × (short axis)]^1/2^ at *t* = 0 (beginning of the fusion). The results showed that *η*/*γ* decreased with decreasing SE length (Fig. 2). Plots of *τ*_fusion_*vs. l* exhibited an approximately linear relationship (Fig. S3†). The values of *η*/*γ* were 26.3, 15.5, 5.5, 3.4, 2.4, 2.0, and 0.9 s μm^−1^ for SE designs of 12 nt, 10 nt, 8 nt, 6 nt_A, 6 nt_B, 6 nt_C, and 4 nt, respectively. Compared with previously reported values measured at 20 °C (19.5, 19.7, and 23.8 s μm^−1^ in 0.25, 0.5, and 1 M NaCl, respectively, for an SE length of 6 nt),^23^ the values obtained for SE designs of 12 nt and 10 nt were similar, whereas those obtained for SE designs of 8 nt, 6 nt_A, 6 nt_B, 6 nt_C, and 4 nt were one or two orders of magnitude smaller.

**Fig. 3:**
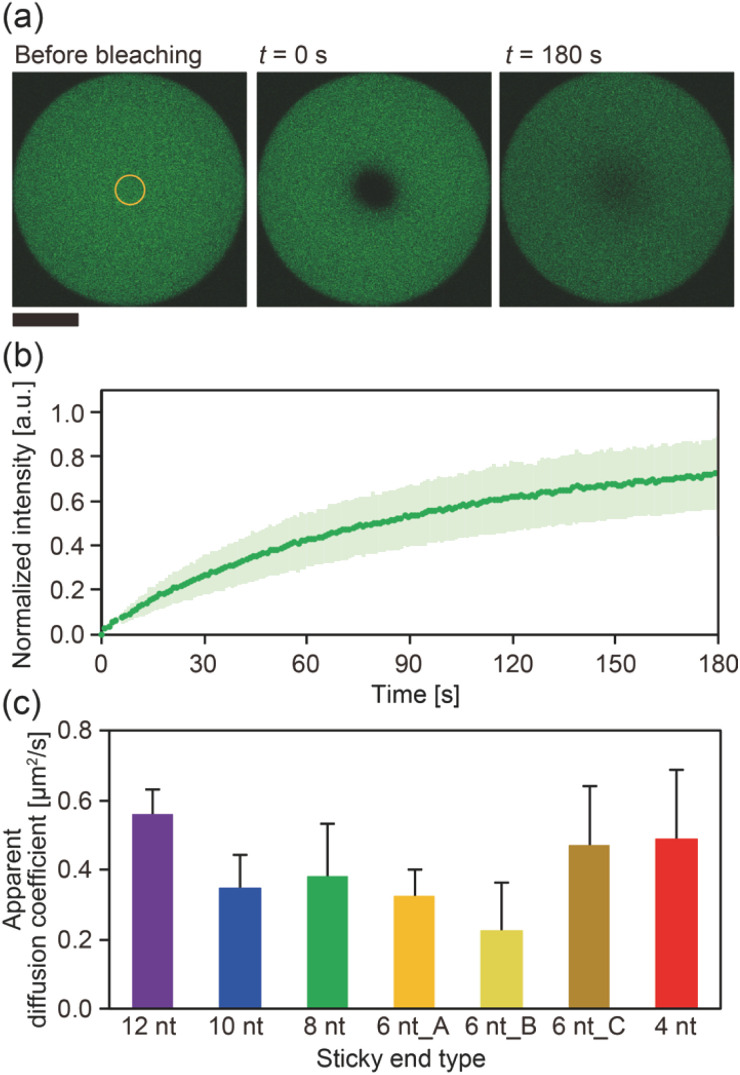
Mobility analysis for the Y-motifs forming the droplets. (a) Representative images of the FRAP experiments for a droplet composed of the Y-motif with an SE length of 8 nt. The yellow circle indicates the region subjected to bleaching. Scale bar: 30 μm. (b) Recovery curve of the fluorescence intensity of the bleached region for a droplet composed of the Y-motif with an SE length of 8 nt. The green line and light green area indicate the average values and standard deviations, respectively (*n* = 9). (c) Apparent diffusion coefficients of the Y-motifs with different SE designs. Error bars indicate standard deviations (*n* = 10, 7, 9, 9, 10, 10, and 6 for SE designs of 12 nt, 10 nt, 8 nt, 6 nt_A, 6 nt_B, 6 nt_C, and 4 nt, respectively). Note that the FRAP experiments were performed at 69, 60, 62, 47, 48, 42, and 45 °C for SE designs of 12 nt, 10 nt, 8 nt, 6 nt_A, 6 nt_B, 6 nt_C, and 4 nt, respectively.

To gain further insight into the SE interactions in the DNA droplets, the Y-motif mobility in the droplets was evaluated by performing FRAP experiments. The centre of the droplets was bleached (Fig. 3a) and the recovery of the fluorescence intensity in the bleached region was recorded (Fig. 3b). From the recovery curve of the fluorescence intensity, we calculated the apparent diffusion coefficient of the Y-motif for each SE design. Although the inverse capillary velocity values were very different (28 times difference between 12 nt and 4 nt designs), such a large difference was not confirmed in the apparent diffusion coefficients at *T*_d_ (Fig. 3c and S4†).

## 4 Discussion

Our experimental results showed that at *T*_d_, (i) the DNA droplets composed of Y-motifs with longer SEs required a longer time (*τ*_fusion_) to complete fusion and (ii) had a higher inverse capillary velocity (*η*/*γ*), but (iii) the mobility of the Y-motifs in the droplets did not strongly depend on the SE design. Here, we discuss how SE design influences the physical properties of the DNA droplets.

Both the viscosity and the surface tension can be altered by the SE behaviour. Several studies have reported the temperature dependence of DNA hydrogel viscosity, which was ascribed to the bond stability of the DNA nanostructures.^30,31^ The *D*_app_ of the motif in the droplet represents the ratio between association and dissociation frequencies of the SEs (Fig. 3c). At the *T*_d_, the bond stability, *i.e.*, the frequency of association and dissociation, was assumed to be similarly very high for all SE designs because the *T*_d_ of each SE is a much higher temperature than *T*_m_. However, the FRAP results indicated that the *D*_app_ was slightly different in each SE design. Consequently, it is hypothesized that the viscosity of the droplets may also vary at their correspondent *T*_d_. This hypothesis was supported by microrheological measurements in which the Y-motifs were used as tracer particles, indicating that the viscosity could be varied in the range of 0.08 to 0.23 Pa s (Fig. S5†).

**Fig. 4:**
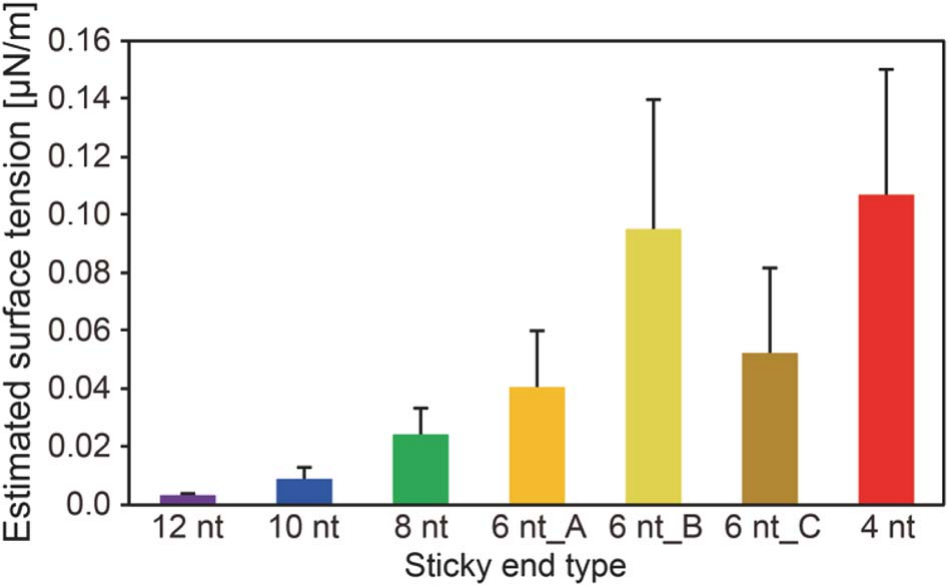
Estimated surface tension of the DNA droplets at *T*_d_. These values were obtained based on the inverse capillary velocity and the estimated viscosity by the FRAP experiments. Error bars were obtained using error propagation theory.

We obtained the surface tension values based on the estimated viscosity and the inverse capillary velocity values (Fig. 4). Here, to obtain the surface tension values, we substituted the estimated viscosity for *η* in the inverse capillary velocity values (*η*/*γ*) (Fig. 2). It was found that the surface tension of the DNA droplets at *T*_d_ tended to decrease with increasing SE length (0.003 μN m^−1^ for 12 nt and 0.11 μN m^−1^ for 4 nt). Jeon *et al.* reported that unbound base pairs on the surface of liquid-like DNA condensates can strongly affect the surface tension.^23^ Our previous work^27^ demonstrated that the concentration of Y-motifs in the DNA droplets was higher for shorter SEs. Thus, the number of free SEs on the droplet surface can become higher for shorter SE lengths. This may account for the differences in surface tension at *T*_d_ among the different SE lengths.

Hairpin structure formation of the SEs would also affect the surface tension. We designed the SE sequences as palindromic; thus, they can form hairpin structures. This should reduce the number of unbound base pairs of the exposed SEs at the interface between the DNA droplets and bulk solution. Numerical analysis revealed that the probability of hairpin formation of the SEs at *T*_d_ tends to increase with increasing SE length, with estimated probabilities of 11.1%, 12.8%, 3.1%, 0%, 0%, 0%, and 0% for SE designs of 12 nt, 10 nt, 8 nt, 6 nt_A, 6 nt_B, 6 nt_C, and 4 nt, respectively (Fig. S6†). It was confirmed that a higher probability of hairpin formation at a lower temperature leads to the lower surface tension values (Fig. S6 and S7†). However, hairpin formation alone cannot explain the differences in the surface tension because SEs shorter than 6 nt cannot form hairpin structures at *T*_d_. Therefore, a combination of factors, including differences in the Y-motif concentration and the hairpin formation probability, appears to affect the surface tension.

We also found that when the number of SEs in the motif increased (from three to four or six), the inverse capillary velocity decreased (5.5, 2.7, and 1.7 s μm^−1^ for three, four, and six SEs, respectively), reflecting the faster fusion dynamics of the DNA droplets (Fig. S8†). Note that the motifs with four and six SEs also exhibited a linear relationship in plots of *τ*_fusion_*vs. l* (Fig. S9†). Increasing the number of SEs can lead to an increased number of free SEs on the droplet surface, which may result in higher surface tension. On the other hand, although the mobility of the Y-motif (three SEs) in the DNA droplets was similar at *T*_d_, an increased number of SEs decreased the motif mobility at *T*_d_,^27^ suggesting an increase in the viscosity. We previously found that *T*_d_ increased with the number of SEs,^27^ which suggests that the collision frequency of the SEs among the motifs is important for the formation of the DNA droplets. The mobility of the motifs in the DNA droplets can be determined by hybridization of the SEs. It is expected that a higher collision frequency of the SEs in the droplets favours duplex formation of the SEs. Therefore, increasing the number of SEs should lead to an increase in the viscosity. In summary, at *T*_d_, the SE design greatly affects the surface tension but not the viscosity, while the number of SEs may influence both.

Consideration of the hybridization thermodynamics may provide a quantitative interpretation. To this end, the hybridization free energy (|Δ*G*|) of the SEs can be used. Jeon *et al.* described the relationship between the physical properties of DNA droplets and the salt concentration, which is one parameter that determines the |Δ*G*| of the SEs.^23^ They proposed that *γ* ∝ |Δ*G*|/*v*_mol_^2/3^ (where *v*_mol_ denotes the volume occupied by one DNA nanostructure) and larger |Δ*G*| exhibited a higher viscosity.^23^ We compared |Δ*G*| at *T*_d_ for all of our SE designs; however, the two order magnitude difference of |Δ*G*|, as we estimated for *γ* (Fig. 4), could not be confirmed (5.0, 5.1, 4.0, 3.3, 3.2, 5.8, and 3.1 kcal mol^−1^ for SE designs of 12 nt, 10 nt, 8 nt, 6 nt_A, 6 nt_B, 6 nt_C, and 4 nt). In addition, the |Δ*G*| differences in different SE designs were not consistent with the trend of the estimated *η* values (Fig. S5†). This may be attributable to the much higher values of *T*_d_ compared with *T*_m_ for the SEs. The previous work was performed at 20 °C, where the hybridization probability exceeded 98% (|Δ*G*| ≈ 9 kcal mol^−1^ at 1 M NaCl).^23^ In contrast, the probability under our experimental conditions was less than 5% for all of the SEs (Fig. S6†). On this basis, only the free energy of single SE hybridization (interaction of two DNA strands) may be insufficient for explaining the interactions of DNA nanostructures with multiple SEs and the dynamics of SE behaviour under such unstable binding conditions.

## 5 Conclusions

Our experimental results revealed that the SE design in the Y-motifs drastically affected the fusion dynamics of the DNA droplets at the phase transition temperature (Fig. 1c), as represented by the inverse capillary velocity. The inverse capillary velocity at *T*_d_ was 26.2 s μm^−1^ for an SE design of 12 nt but decreased to only 0.9 s μm^−1^ for an SE design of 4 nt (Fig. 2). FRAP experiments revealed that the mobility of the Y-motif was varied by the SE design (Fig. 3), suggesting that the DNA droplets possessed different viscosities at *T*_d_. The surface tension of the DNA droplets significantly increased with decreasing SE length (Fig. 4). These results may be explained by concentration differences of the Y-motif inside the droplets depending on the SE length and hairpin formation by the SEs. We adopted the *T*_d_ for each design SE as a measurement temperature to focus on the fusion dynamics. It should be noted that the measurement temperature can influence the physical properties (Fig. S7†). In addition, salt concentration^23^ and structural design, such as flexibility of SEs,^28^ will affect the fusion behaviour of the DNA droplets. Further quantitative analysis will be required to comprehensively elucidate the underlying mechanisms determining the physical properties of DNA droplets.

An understanding of the SE-design-dependent differences in these physical properties should broaden the applications of DNA-based LLPS systems. The stability of DNA hybridization can be altered by not only changing the salt concentration and solution temperature but also photo-irradiation, *e.g.*, after modification with photoresponsive molecules such as azobenzene.^37^ A similar concept was demonstrated for the stimuli-responsive assembly/disassembly of LLPS droplets.^38,39^ Because the physical properties of LLPS droplets in cells play a key role in their functions as chemical reactors or mechanical actuators,^2^ the photoresponsive changes of physical properties should enable the construction of stimuli-responsive functional liquid-like condensates with features such as membrane deformation capability,^4^ phototaxis-like motion,^40^ and computation.^41^ Such macromolecular structures are expected to prove useful for the construction of functional artificial cells or molecular robots using biopolymer-based LLPS systems.

## Author contributions

Conceptualization: Y. S. and M. T. Investigation: Y. S. Resources: Y. S. and M. T. Writing – original draft: Y. S. Writing – review & editing: Y. S. and M. T.

## Conflicts of interest

There are no conflicts to declare.

## Supplementary Material

NA-005-D3NA00073G-s001
