# Supplementary material for: Sequence-dependent fusion dynamics and physical properties of DNA droplets
Source: Nanoscale Adv. 2023 Feb 14;5(7):1919–25. doi: 10.1039/d3na00073g (PMC10044877; doi:10.1039/d3na00073g)
Supplement: NA-005-D3NA00073G-s001 [file NA-005-D3NA00073G-s001.pdf]

Electronic Supplementary Material (ESI) for Nanoscale Advances.
This journal is © The Royal Society of Chemistry 2023

Supplementary Information for

Sequence-dependent fusion dynamics and physical properties of DNA droplets

Yusuke Sato,*[a, b] and Masahiro Takinoue*[a]

[a] Department of Computer Science, Tokyo Institute of Technology, Kanagawa 226-8502, Japan
[b] Department of Intelligent and Control Systems, Kyushu Institute of Technology, Fukuoka 820-8502, Japan
Email: ysato@ics.kyutech.ac.jp (Y.S), takinoue@c.titech.ac.jp (M.T)

This PDF file includes:

Table S1: DNA sequences
Figure S1: Sequential images of the fusion process for DNA droplets composed of the various Y-motifs
Figure S2: Time course of the aspect ratios of the DNA droplets composed of the Y-motifs with different sticky end lengths
Figure S3: Plots of τ_{fusion} *vs.* characteristic length *l*
Figure S4: Fluorescence recovery after photobleaching of the DNA droplets composed of the Y-motifs with different sticky end lengths
Figure S5: Estimated viscosities based on microrheological measurements
Figure S6: Hybridization and hairpin formation probabilities of the sticky ends
Figure S7: Comparison of the physical properties of the DNA droplets composed of the Y-motif with 8 nt SE at different temperatures
Figure S8: Effects of the number of sticky ends on the fusion dynamics and physical properties of the DNA droplets
Figure S9: Plots of τ_{fusion} *vs.* characteristic length *l* for different numbers of sticky ends (8 nt in length) in the motif

Table S1: DNA sequences

Name	Sequence (5′–3′)
Y-1_12	GCTCGAGCCAGTGAGGACGGAAGTTTGTCGTAGCATCGCACC
Y-2_12	GCTCGAGCCAACCACGCCTGTCCATTACTTCCGTCCTCACTG
Y-3_12	GCTCGAGCGGTGCGATGCTACGACTTTGGACAGGCGTGGTTG
Y-1_10	GACTCGAGTCCAGTGAGGACGGAAGTTTGTCGTAGCATCGCACC
Y-2_10	GACTCGAGTCCAACCACGCCTGTCCATTACTTCCGTCCTCACTG
Y-3_10	GACTCGAGTCGGTGCGATGCTACGACTTTGGACAGGCGTGGTTG
Y-1_8	GCTCGAGCCAGTGAGGACGGAAGTTTGTCGTAGCATCGCACC
Y-2_8	GCTCGAGCCAACCACGCCTGTCCATTACTTCCGTCCTCACTG
Y-3_8	GCTCGAGCGGTGCGATGCTACGACTTTGGACAGGCGTGGTTG
Y-1_6_A	GCTAGCCAGTGAGGACGGAAGTTTGTCGTAGCATCGCACC
Y-2_6_A	GCTAGCCAACCACGCCTGTCCATTACTTCCGTCCTCACTG
Y-3_6_A	GCTAGCGGTGCGATGCTACGACTTTGGACAGGCGTGGTTG
Y-1_6_B	GTCGACCAGTGAGGACGGAAGTTTGTCGTAGCATCGCACC
Y-2_6_B	GTCGACCAACCACGCCTGTCCATTACTTCCGTCCTCACTG
Y-3_6_B	GTCGACGGTGCGATGCTACGACTTTGGACAGGCGTGGTTG
Y-1_6_C	TGCGCACAGTGAGGACGGAAGTTTGTCGTAGCATCGCACC
Y-2_6_C	TGCGCACAACCACGCCTGTCCATTACTTCCGTCCTCACTG
Y-3_6_C	TGCGCAGGTGCGATGCTACGACTTTGGACAGGCGTGGTTG
Y-1_4	GCGCCAGTGAGGACGGAAGTTTGTCGTAGCATCGCACC
Y-2_4	GCGCCAACCACGCCTGTCCATTACTTCCGTCCTCACTG
Y-3_4	GCGCGGTGCGATGCTACGACTTTGGACAGGCGTGGTTG
Four-1_8	GCTCGAGCGCTGGACTAACGGAACGGTTAGTCAGGTATGCCAGCAC
Four-2_8	GCTCGAGCGTGCTGGCATACCTGACTTTCGCAAATTTACAGCGCCG
Four-3_8	GCTCGAGCCGGCGCTGTAAATTTGCGTTCATCACTTGGGACCATGG
Four-4_8	CTCGCGAGCCATGGTCCCAAGTGATGTTCCGTTCCGTTAGTCCAGC
Six-1_8	GCTCGAGCGCTGGACTAACGGAACGGTTAGTCAGGTATGCCAGCAC
Six-2_8	GCTCGAGCCTCAGAGAGGTGACAGCATTCCGTTCCGTTAGTCCAGC
Six-3_8	GCTCGAGCCCATGGTCCCAAGTGATGTTTGCTGTCACCTCTCTGAG
Six-4_8	GCTCGAGCCGGCGCTGTAAATTTGCGTTCATCACTTGGGACCATGG
Six-5_8	GCTCGAGCCAGACGTCACTCTCCAACTTCGCAAATTTACAGCGCCG
Six-6_8	GCTCGAGCGTGCTGGCATACCTGACTTTGTTGGAGAGTGACGTCTG
Y-2_0_FAM	[6-FAM]-CAACCACGCCTGTCCATTACTTCCGTCCTCACTG
Four-2_0_Cy3	[Cy3]-GTGCTGGCATACCTGACTTTCGCAAATTTACAGCGCCG
Six-2_0_Cy5	[Cy5]-CAGACGTCACTCTCCAACTTCGCAAATTTACAGCGCCG

‡ Black-coloured bases indicate the sticky end parts. Grey-coloured bases indicate the flexible single-stranded region. Other-coloured bases indicate the complementary regions.

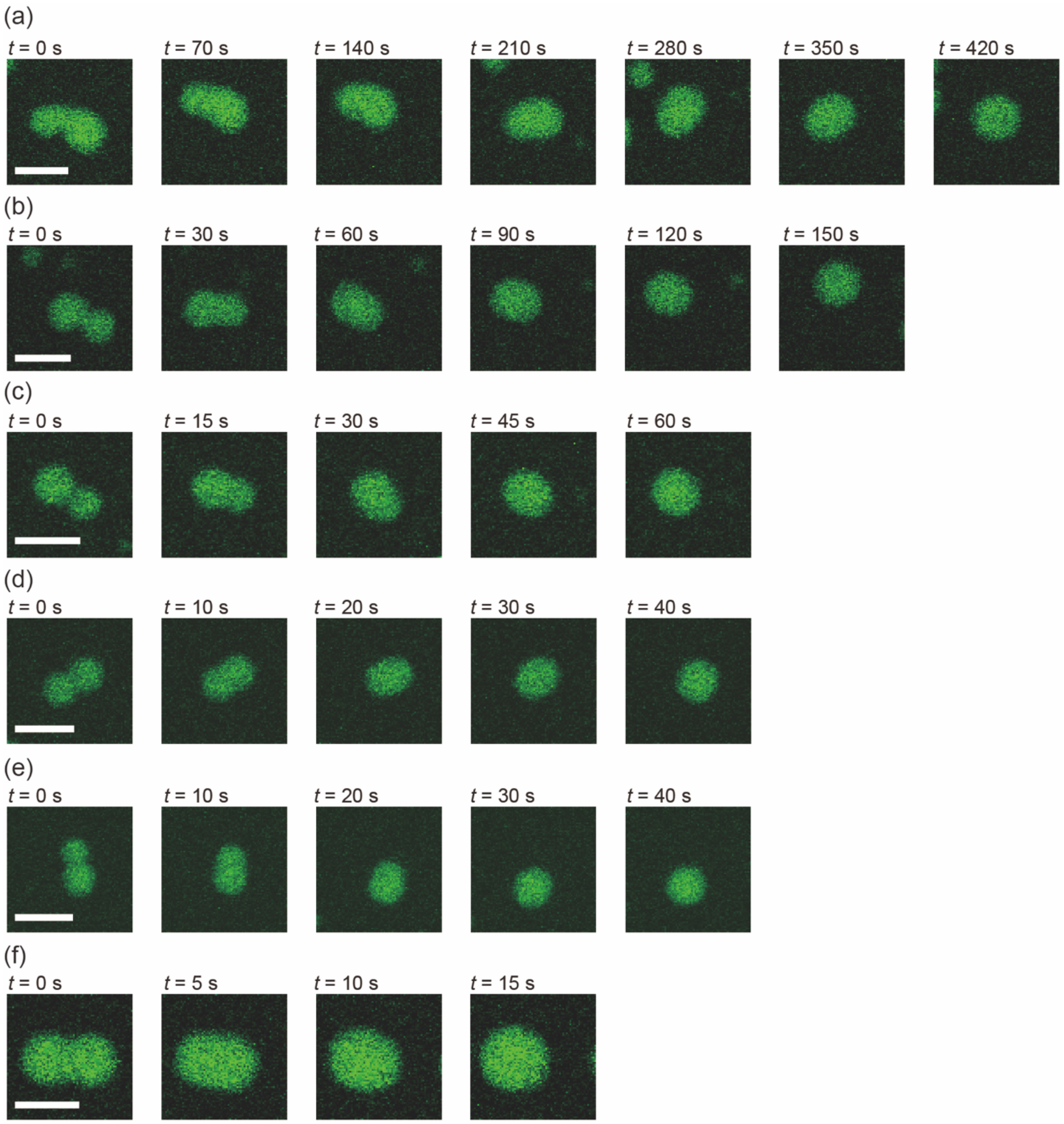

Figure S1. Representative sequential images of the fusion process for DNA droplets composed of the Y-motifs with SE designs of (a) 12 nt, (b) 10 nt, (c) 6 nt_A, (d) 6 nt_B, (e) 6 nt_C, and (f) 4 nt. The corresponding aspect ratio changes are shown in Fig. 1c in the main text. Scale bars: 10 µm.

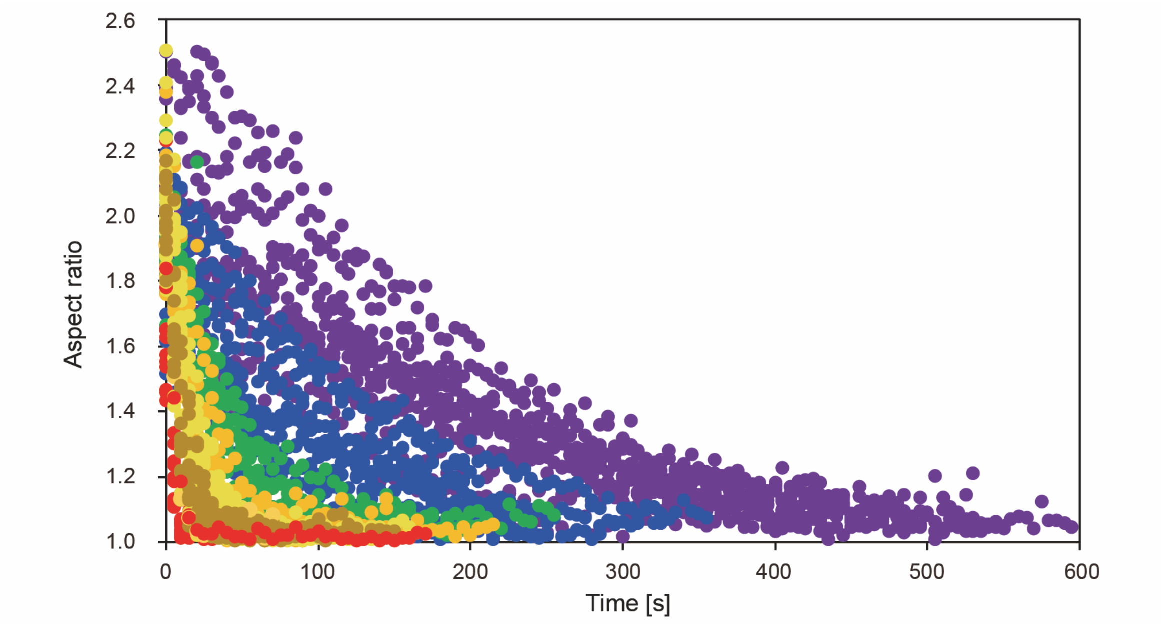

Figure S2. Time course of the aspect ratios of the DNA droplets composed of the Y-motifs with different SE designs. The purple, blue, green, yellow, light yellow, dark yellow, and red colours correspond to SE designs of 12 nt, 10 nt, 8 nt, 6 nt_A, 6 nt_B, 6 nt_C, and 4 nt, respectively.

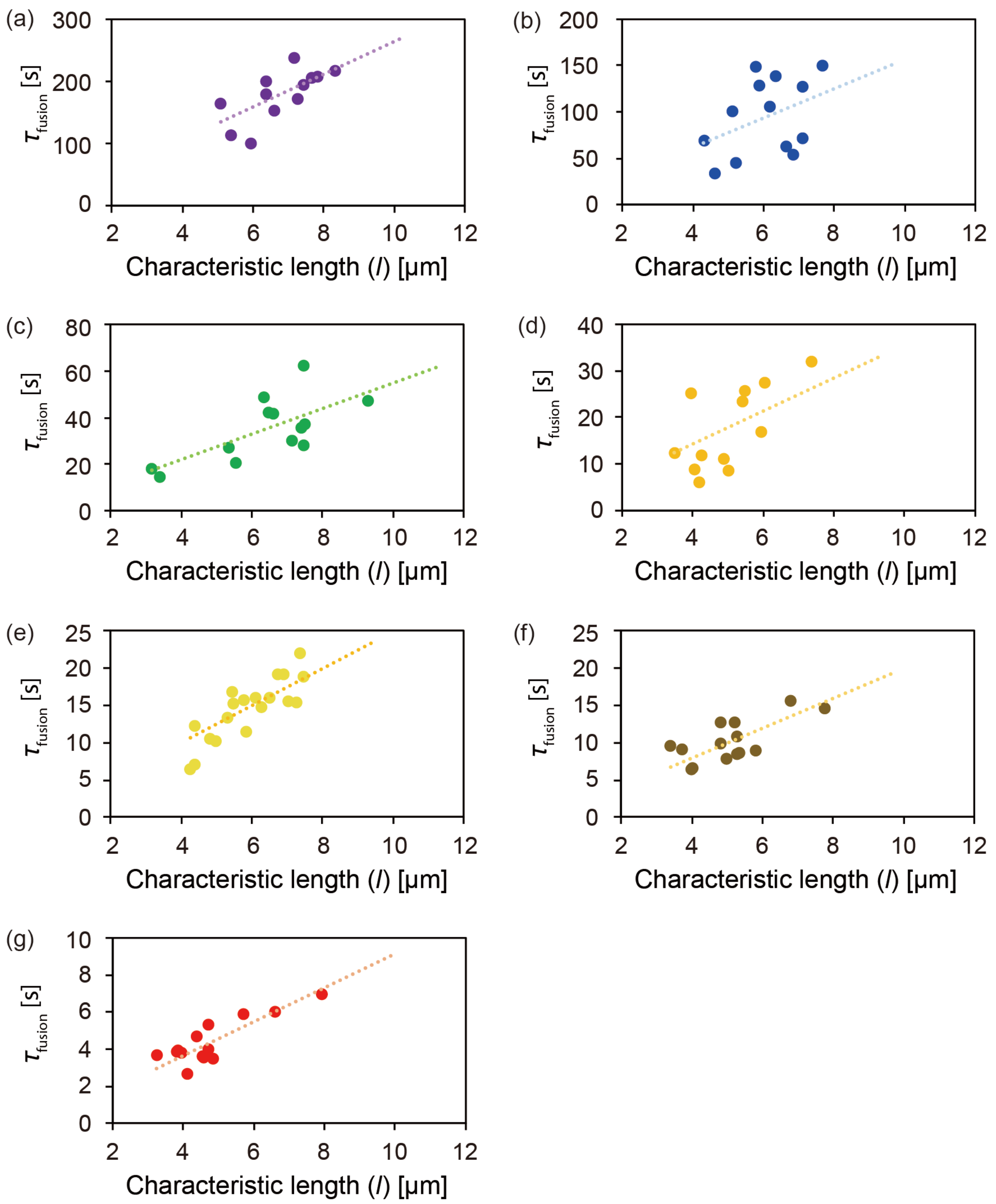

Figure S3. Plots of τ_{fusion} *vs.* characteristic length *l* for SE lengths of (a) 12 nt, (b) 10 nt, (c) 8 nt, (d) 6 nt_A, (e) 6 nt_B, (f) 6 nt_C, and (g) 4 nt. The closed circles represent experimental values and the dashed lines show linear fittings as a guide for the eye.

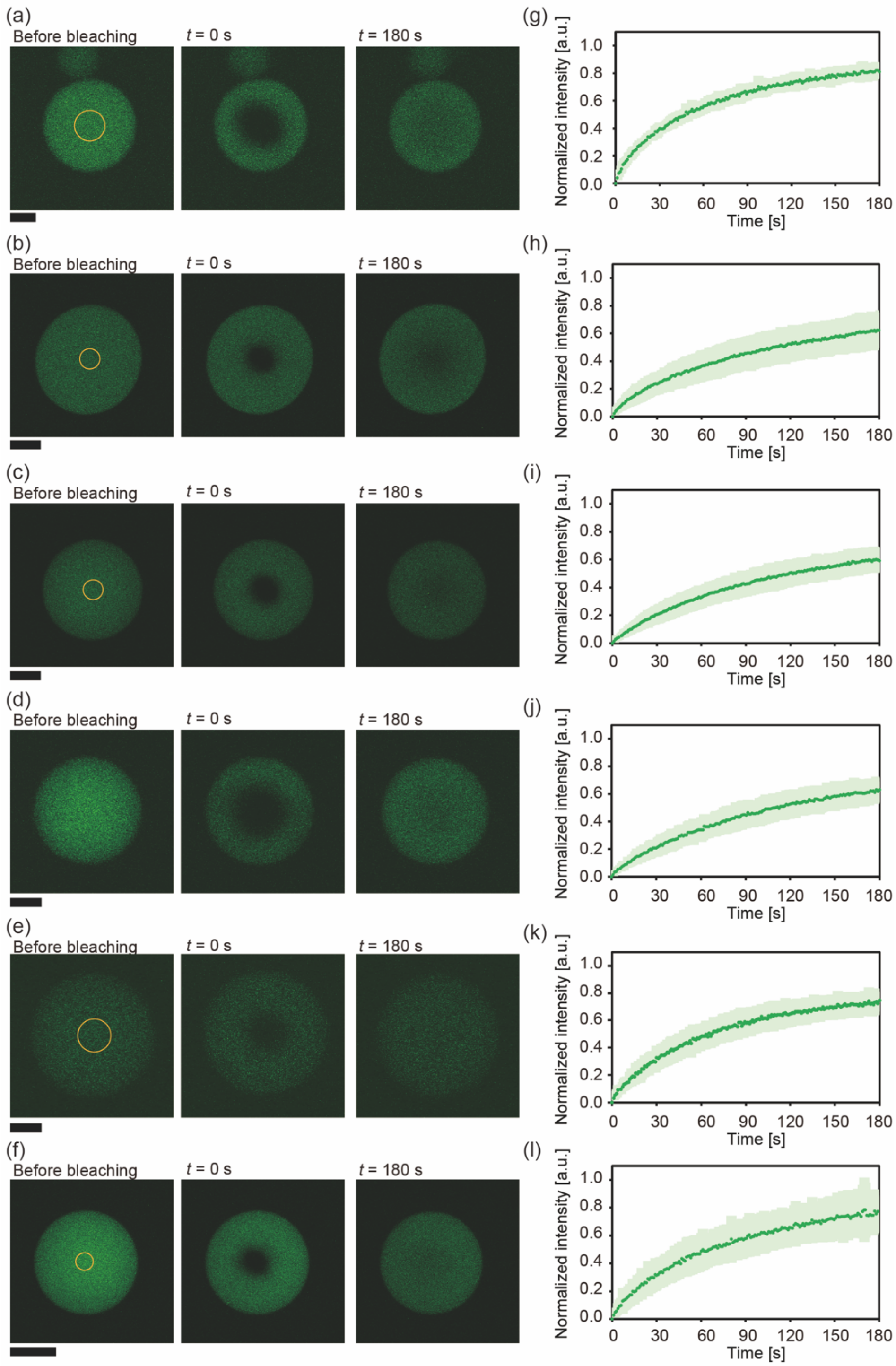

Figure S4. Fluorescence recovery after photobleaching results for the DNA droplets composed of the Y-motifs with SE lengths of (a) 12 nt, (b) 10 nt, (c) 6 nt_A, (d) 6 nt_B, (e) 6 nt_C, and (f) 4 nt. Scale bars: 10 μm in (a), (d), and (e), 20 μm in (b) and (c), and 30 μm in (f). (g)–(l) Corresponding recovery curves of the fluorescence intensity of the bleached region. The green lines and light green areas indicate the average values and standard deviations, respectively.

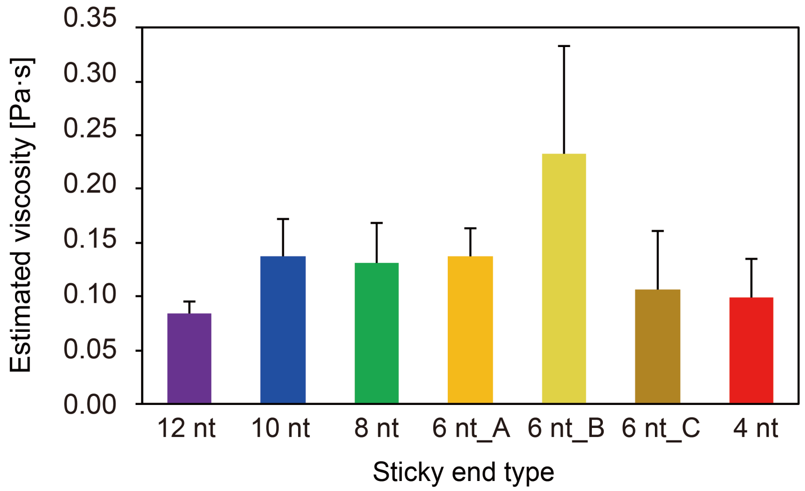

Figure S5. Estimated viscosities based on microrheological measurements. The Y-motifs were used as tracer particles. Here, it was assumed that the Y-motifs had a hydrodynamic radius of 5.44 nm, considering the distance per base pair (0.34 nm/bp), persistence length of double-stranded DNA (dsDNA) and single-stranded DNA (ssDNA), and the flexible joints at the centres of the motifs. Each Y-motif has 16-base-pair dsDNA stems and 4–12 nt single-stranded SEs. We hypothesized that the influence of the SE length on the hydrodynamic radius was negligible. Using the apparent diffusion coefficient values (Fig. 3c in the main text), the aforementioned hydrodynamic radius, and the Stokes–Einstein relation ($D=k_BT/6\pi\eta R$), the viscosity was estimated.

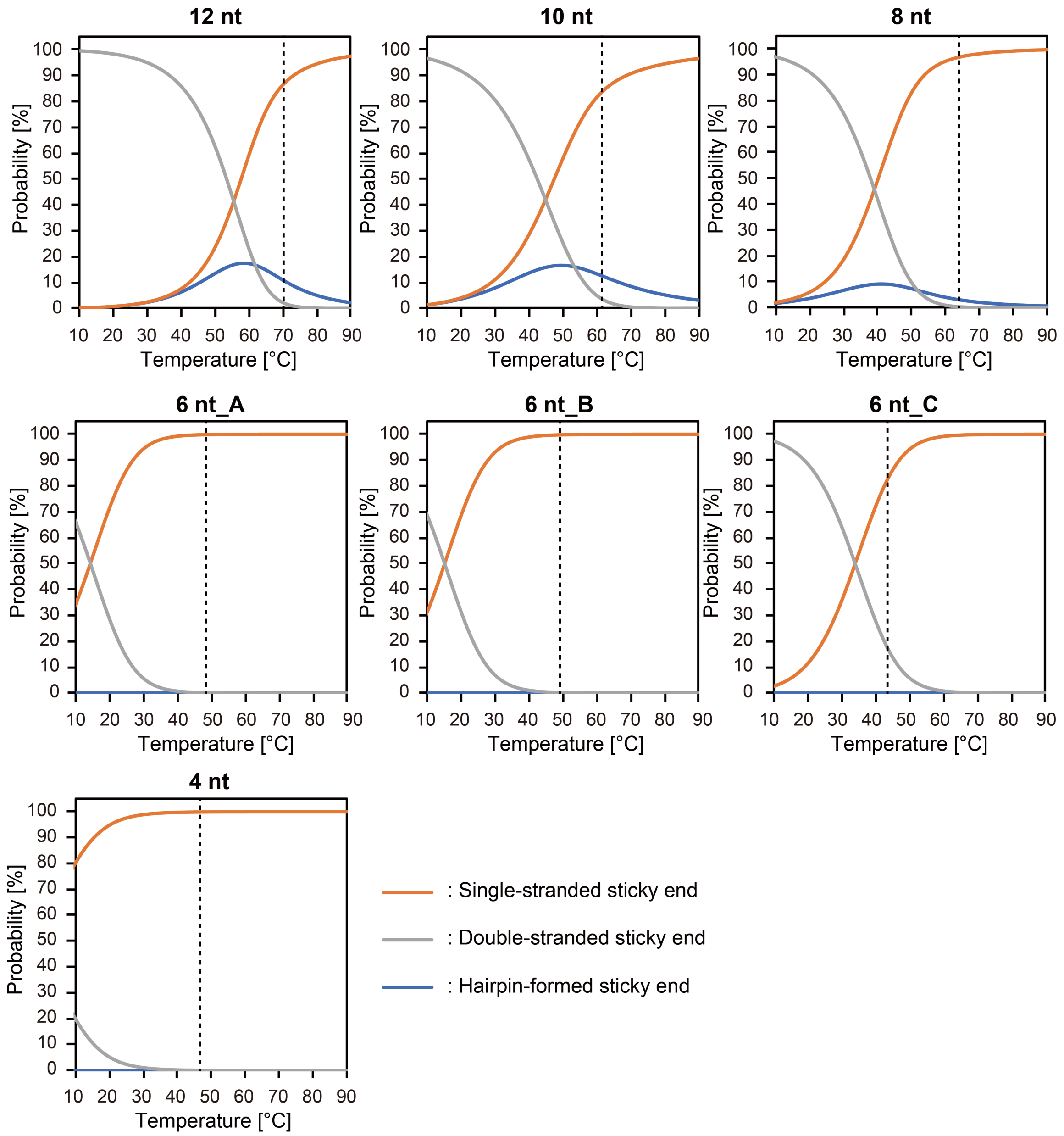

Figure S6. Hybridization and hairpin formation probabilities of the SEs. The vertical dashed lines in each plot indicate the T_d.

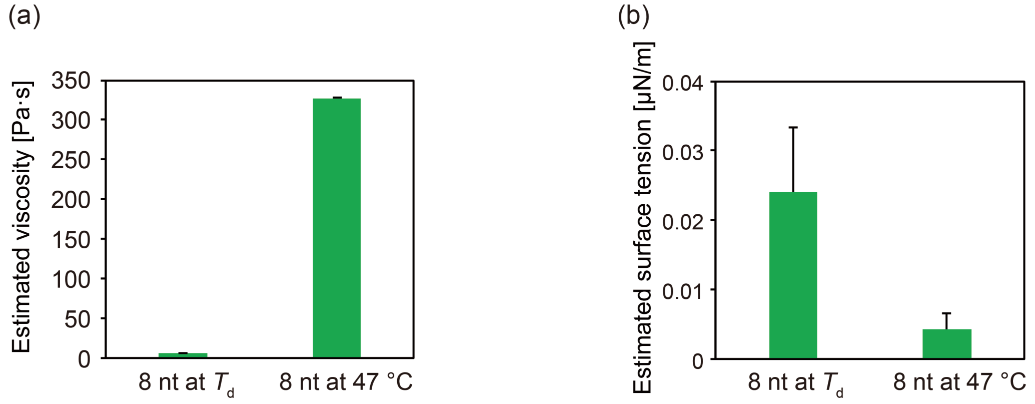

Figure S7. Comparison of the physical properties of the DNA droplets composed of the Y-motif with 8 nt SE at different temperatures. (a) Estimated viscosity. (b) Estimated surface tension. The method to obtain these values was the same as described the main text.

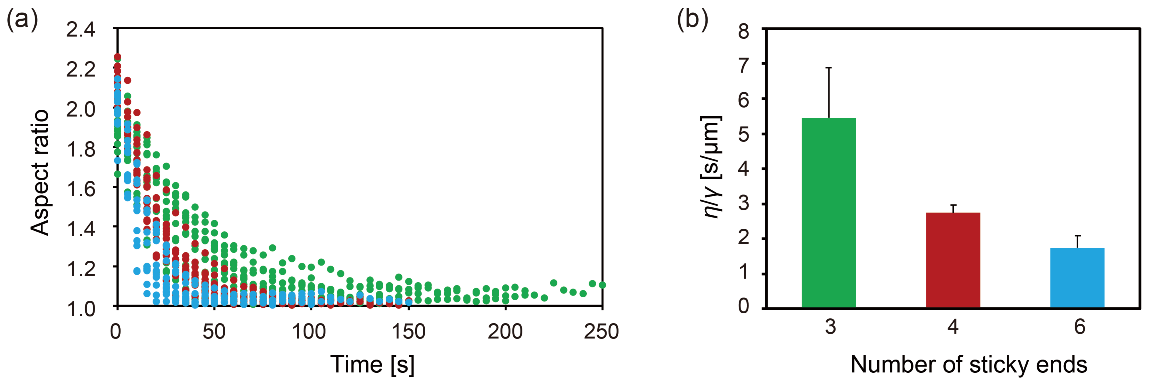

Figure S8. Effects of the number of SEs on the fusion dynamics and physical properties of the DNA droplets. (a) Aspect ratio changes over time for the fusion of DNA droplets composed of the DNA nanostructures with four or six SEs (8 nt in length). The green, dark red, and cyan closed circles correspond to three, four, and six SEs in the motif, respectively. These data were obtained at T_d (63.7±0.6 °C for three SEs, 67.7±0.6 °C for four SEs, and 72.3±1.2 °C for six SEs).[27] (b) Inverse capillary velocities of DNA droplets composed of Y-motifs with various SE lengths at T_d. Error bars indicate standard deviations (n = 13, 14, and 12 for three, four, and six SEs, respectively).

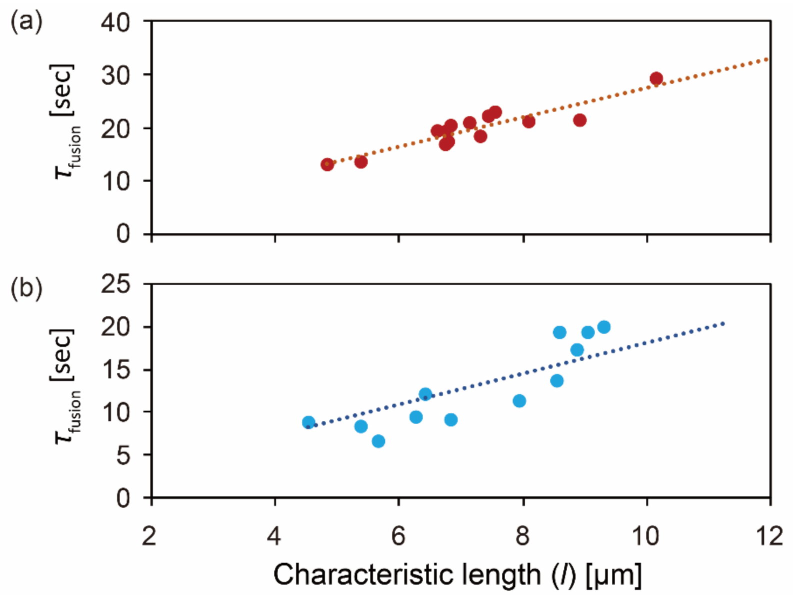

Figure S9. Plots of τ_{fusion} *vs.* characteristic length *l* for (a) four and (b) six SEs (8 nt in length) in the motif. The closed circles represent experimental values and the dashed lines show linear fittings as a guide for the eye.
